# A Review of Yellow Fever: Epidemiology, Pathogenesis, Clinical Manifestations, Treatment, Prevention, and Prognosis

**DOI:** 10.7759/cureus.106825

**Published:** 2026-04-10

**Authors:** Farah Ebrahim, Samina Somji, Reena Shah, Salim Surani

**Affiliations:** 1 Medicine, Aga Khan University Hospital, Nairobi, KEN; 2 Internal Medicine, Aga Khan University Medical College, Dar es Salaam, TZA; 3 Medicine, Aga Khan University, Nairobi, KEN; 4 Medicine, University of Houston, Houston, USA; 5 Anesthesiology, Mayo Clinic, Rochester, USA

**Keywords:** aedes, mosquito-borne, vaccination, yellow fever, yellow fever virus

## Abstract

Yellow fever is a mosquito-borne viral illness caused by the yellow fever virus (YFV), a member of the *Flaviviridae* family. Despite the availability of an effective live-attenuated vaccine, the disease remains endemic in regions of Africa and South America. Transmission occurs primarily via an infected mosquito called *Aedes aegypti*, with distinct urban, sylvatic, and intermediate transmission cycles. Clinically, infection ranges from mild febrile illness to severe disease, which is characterized by jaundice, hemorrhage, and multiorgan failure. Pathogenesis involves viral replication in hepatocytes, immune-mediated injury, and dysregulated inflammatory responses, leading to characteristic hepatic mid-zone necrosis. Diagnosis relies on molecular detection of viral RNA, serological testing, and supporting laboratory findings. Management remains largely supportive, as no antiviral therapy is available. Preventive strategies focus on vaccination and vector control, and surveillance, which are limited during outbreaks, reduce mortality. Yellow fever continues to be a neglected disease despite the availability of an effective vaccine. Improved vaccination coverage and enhanced surveillance systems are essential to prevent its re-emergence in endemic areas.

## Introduction and background

Yellow fever first emerged in Africa around 3,000 years ago, with early outbreaks in West African populations. Yellow fever was subsequently introduced in the Americas through the transatlantic slave trade, where it established a sylvatic transmission cycle in the rainforests of Central and South America [1]. The etiologic agent remained unknown until the early 20th century, when Dr. Walter Reed and the U.S. Army Yellow Fever Commission demonstrated in 1900 that transmission occurs via the mosquito *Aedes aegypti* [1]. Advances in vaccine development followed in the first half of the 20th century, particularly after the isolation of the Asibi strain in Nigeria in 1927, which Max Theiler and colleagues attenuated to produce the 17D vaccine strain, which remains the basis of current yellow fever immunization programs [2]. 

The transmission of pathogens to humans through insect vectors has long involved animal hosts. A classic example of vector-borne transmission is the yellow fever virus (YFV), which is transmitted to humans by mosquitoes [3,4].

Infection typically occurs through the bite of an infected *Ae. aegypti* mosquito, a species widely distributed in tropical and subtropical regions [3-5]. Yellow fever transmission involves two distinct cycles: an urban cycle, in which outbreaks are less frequent but can be highly lethal when they occur, and a sylvatic cycle (primarily among non-human primates), which accounts for most transmission [3]. The virus was first isolated in a male patient in 1927 [6]. Yellow fever is a flavivirus that causes acute infection and may be life-threatening and remains endemic in tropical regions of Africa and South America [7,8]. Although an effective vaccine has been available since 1937, and vaccination campaigns and immunization programs have been widely implemented, significant outbreaks continue to occur in sub-Saharan Africa [8]. Between December 2016 and June 2017, Brazil experienced a major yellow fever outbreak across 21 states, resulting in 777 confirmed cases and 261 deaths [9]. The yellow fever outbreak in Angola, which began in December 2015, lasted until December 2016. In 2016, the outbreak spread to Kinshasa, Democratic Republic of Congo, and extended internationally to countries including Kenya and China [10]. In Kenya, yellow fever outbreaks have been documented since 1992, with the most recent occurring in 2022 in Isiolo County, where 11 cases were reported, including six fatalities [5]. In 2023, six confirmed cases were identified in the Central African Republic, a country recognized by the World Health Organization (WHO) as a high-risk country for yellow fever [8]. Overall, sub-Saharan Africa carries approximately 90% of the global yellow fever burden.

The clinical presentation of yellow fever includes pyrexia and jaundice of the skin and sclera, with symptoms ranging from mild illness to severe hemorrhagic disease [11]. Common manifestations include fever, headache, muscle pain, nausea, vomiting, fatigue, and gastrointestinal symptoms [6]. This wide clinical spectrum complicates diagnosis. In severe cases, hemorrhagic fever may develop and can be fatal.

Yellow fever control depends largely on the highly effective live attenuated 17D vaccine [12]; however, transmission has resurged since the mid-2000s, resulting in recurrent outbreaks [7]. Despite causing an estimated 81,000 deaths annually worldwide, yellow fever remains a neglected disease [5]. This review summarizes the epidemiology, endemicity, pathogenesis, clinical manifestations, prevention, and treatment of yellow fever.

## Review

Epidemiology

Yellow fever is transmitted by day-biting mosquitoes (*Ae. aegypti*). A global resurgence of yellow fever has been observed in the last two decades, influenced by reduced population immunity, environmental changes, urbanization, migration, and climate change [4,13]. The disease is endemic in 47 countries, including 34 in Africa and 13 in Central and South America [4,7].

Angola is an endemic country and experienced its largest outbreak between late 2015 and December 2016 [10]. The outbreak originated in the densely populated capital, Luanda, and rapidly spread nationwide, with subsequent international transmission to the Democratic Republic of the Congo, Kenya, and mainland China. Angola reported 884 laboratory-confirmed cases and 121 deaths. During the same period, the Democratic Republic of the Congo reported an outbreak from July 2016 to February 2017, with 81 laboratory-confirmed cases and 16 deaths [6].

In Brazil, a yellow fever outbreak occurred between 2002 and 2003, with 63 confirmed cases and 23 deaths. This was followed by a larger outbreak between December 2016 and April 2017, during which 623 cases and 326 deaths were reported [3,9].

The Ministry of Health in Kenya reported a yellow fever outbreak in March 2022, with 53 cases and six deaths recorded in Isiolo County [5,14,15]. Although Kenya had experienced imported cases linked to the Angola outbreak in 2016, the overall disease burden was likely underestimated due to underreporting.

YFV transmission mirrors its geographic distribution and is shaped by urbanization and habitat loss. Urban expansion and increased human-wildlife interaction sustain viral circulation in non-human primates, raising the risk of outbreaks [16]. In urban areas, *Ae. aegypti* breed in stagnant water in containers like discarded tires, flowerpots, and water cisterns, while in rural areas, they breed in tree holes, leaf axils, and ground puddles [16]. In South America, yellow fever transmission is mainly concentrated in the Amazon Basin, with temperature, rainfall, and altitude influencing its distribution and transmission dynamics [13,17].

Yellow fever microbiology and transmission

The YFV is a single-stranded, positive-sense RNA virus of the genus *Flavivirus* within the family *Flaviviridae* [4,18]. It was the first arthropod-borne virus identified in humans and serves as the prototype of its genus. The virion is an enveloped, spherical particle about 50 nm in diameter, featuring an icosahedral nucleocapsid and surface projections [4]. In the early 1800s, yellow fever transmission was initially attributed to waterborne spread and direct human contact before the *Ae. aegypti* mosquito was identified as the primary vector [19]. *Ae. aegypti*, the main yellow fever vector, is highly anthropophilic and thrives near humans, especially in urban and suburban areas [19]. *Ae. aegypti* and related species are day-biting, with peak activity in the early morning and late afternoon. *Ae. aegypti* is endophilic, seeking shelter inside houses, and endophagic, feeding indoors, though it also moves between indoor and outdoor environments. It is recognized as a highly competent vector for multiple arboviruses, including dengue, chikungunya, Zika, yellow fever, and Mayaro. Native to Africa, ancestral populations of *Ae. aegypti* still breed in forests and ecotones, with larvae developing in tree holes and adults preferring non-human hosts for blood meals [20].

YFV persists in nature through transovarial transmission (TOT) among mosquitoes, thereby maintaining the virus across mosquito generations [4]. YFV transmission occurs via three main epidemiological cycles: (i) sylvatic (wild) cycle: non-human primates act as the primary reservoir, and humans become infected when entering forested areas, representing most cases in South America; (ii) intermediate (semi-domestic) cycle: occurring at the forest-human settlement interface, particularly in Africa, a “zone of emergence” forms in the savannah during the rainy season, where spillover from sylvatic vectors to humans can occur. This can lead to sustained urban transmission via *Ae. aegypti*; (iii) urban (domestic) cycle: viremic individuals returning from sylvatic or intermediate areas can introduce YFV into densely populated urban settings with high mosquito densities, enabling human-to-mosquito-to-human transmission and potentially large, uncontrolled outbreaks.

Notably, infected individuals are contagious to mosquitoes shortly before symptom onset and for up to five days after fever begins, facilitating further spread [21].

Pathogenesis: viral replication, host immune response, and mechanism of organ damage

Yellow fever is characterized by viral replication, dysregulated host immune responses, and progressive multiorgan injury (the liver being the primary site). Understanding the pathogenesis at a cellular level will provide essential insight into the clinical manifestations and therapeutics involved.

After transmission via the bite of an infected *Ae. aegypti* mosquito, the virus enters the bloodstream and spreads to multiple organs, including the liver, spleen, heart, and kidneys, where it replicates [22]. The lipid envelope, taken from the host cell membrane, carries two glycoproteins, envelope (E) and membrane (M), that facilitate viral attachment and entry into host cells [18]. The viral glycoprotein mediates attachment and entry of the virus into host cells. The envelope (E) glycoprotein promotes fusion of the viral and host cell membranes, enabling release of the nucleocapsid into the cytoplasm, where viral genome translation and replication proceed [23,24]. The structural proteins (C, M/M, and E) form the virion particles, whereas the non-structural proteins (NS1, NS2A, NS2B, NS3, NS4A, NS4B, and NS5) are involved in viral replication and immune evasion. Viral entry and replication involve complex interactions with host cells, during which the virus alters pathogen recognition pathways, stress granule dynamics, and intracellular membrane architecture to support critical stages of its life cycle [23,24]. Within the group of non-structural proteins, NS1 acts as a glycoprotein integral to viral replication and immune evasion. NS2A and NS2B work synergistically to support RNA replication and virion assembly. NS3 exhibits critical enzymatic functions, including protease, helicase, and NTPase activities, that drive viral replication. NS4A and NS4B contribute to RNA replication while modulating host immune responses [25]. NS5, the largest and most conserved non-structural protein, orchestrates viral RNA synthesis through its RNA-dependent RNA polymerase and methyltransferase activities. The primary site of viral antigen detection in severe disease occurs in the liver, predominantly in Kupffer cells [23,24].

The host immune response is a key determinant of the clinical outcome of viral infection. Among immune mechanisms, the humoral response has been the most thoroughly characterized [22]. IgM antibodies are detectable in the first week of illness, reach peak levels during the second week, and then decline rapidly. Early in the illness, circulating cytokine concentrations rise and peak shortly before the terminal phase, reflecting an inflammatory cascade that contributes to multiorgan dysfunction [4]. Significant lymphopenia occurs before biochemical signs of liver injury, indicating early systemic immune dysregulation that may permit unchecked viral replication and ultimately result in immune-mediated tissue damage [4,24]. Neutralizing antibodies serve as the principal mechanism of protection against subsequent reinfection [22].

Analyses of adaptive immune responses in fatal human liver specimens reveal a predominantly CD4⁺ lymphocytic infiltrate, along with increased expression of transforming growth factor beta (TGF-β), supporting the involvement of cytotoxic and cytokine-mediated apoptotic pathways in hepatocyte injury [4]. The interplay between protective antiviral immunity and harmful immune-mediated tissue damage appears to be a key determinant of disease severity.

Organ injury in yellow fever is multifactorial, involving direct viral cytopathic effects, immune-mediated damage, and indirect systemic consequences [4,22,24]. Direct viral injury leads to midzonal (zone 2) hepatocellular apoptosis, histologically characterized by Councilman bodies. Immune-mediated apoptotic pathways, along with hypoxic injury driven by pro-apoptotic signaling, contribute to extensive hepatocyte loss [4,22]. Indirect systemic effects, including coagulopathy, hypovolemia, and cytokine-mediated endothelial dysfunction, further promote multiorgan injury and failure, particularly affecting the heart and kidneys [22].

Clinical manifestation

The clinical course of yellow fever follows a characteristic temporal pattern with variable severity, ranging from an asymptomatic or self-limited febrile illness to fulminant multiorgan failure with high case fatality rates. After the bite of an infected mosquito, the incubation period is typically three to six days [22,23].

The illness typically progresses through three phases: (i) acute febrile phase (Table 1): begins abruptly with high fever, severe headache, myalgia, back pain, nausea, and vomiting, sometimes showing relative bradycardia (Faget sign). Active viral replication occurs during this three- to four-day phase [4,23]. (ii) Remission phase (Table 1): a short recovery period lasting a few hours to two days, during which symptoms improve or temporarily disappear, suggesting apparent recovery [4,23]. (iii) Intoxication phase (Table 1): about 15%-20% of patients who initially improve enter this severe phase, marked by recurrent high fever, worsening jaundice, hepatitis, bleeding, kidney failure, and shock. This stage has the highest mortality and indicates severe viscerotropic disease [4,23,25,26].

**Table 1:** Clinical manifestation Source: [4,23,26] AST: Aspartate aminotransferase; ALT: Alanine aminotransferase; PT: Prothrombin time; INR: International normalized ratio

Phase	Duration	Clinical Features	Frequency	Laboratory Findings
Incubation	3-6 days	Asymptomatic	100%	Normal
Acute febrile phase	3-4 days	High fever (39-40°C), Severe headache, Myalgia (especially back pain), Nausea and vomiting, Facial flushing, Conjunctival injection, Relative bradycardia (Faget sign)	100% of symptomatic	Leukopenia, Lymphopenia, Viremia detectable, Mild transaminase elevation
Remission phase	Hours to 2 days	Symptom improvement, Apparent recovery, May be absent in severe cases	85% (brief recovery)	Declining viremia, Rising antibodies
Intoxication phase	3-8 days	Recurrent high fever, jaundice (hallmark), hemorrhagic manifestations including hematemesis (black vomit), melena, gingival bleeding, petechiae and ecchymoses, oliguria or anuria, hypotension or shock, altered mental status, seizures (rare), coma	15-20% of cases	AST/ALT >1000 IU/L, Hyperbilirubinemia (>5 mg/dL), Prolonged PT/INR, Thrombocytopenia, Elevated creatinine, Metabolic acidosis, Hypoglycemia
Recovery or death	Variable	Gradual resolution (survivors), Multi-organ failure (fatal cases)	Recovery: 80-85%, Death: 20-50% of severe cases	Normalizing parameters (survivors), Progressive organ failure (fatal)

Severe yellow fever is characterized by hepatic dysfunction with marked transaminase elevation and coagulopathy. Aminotransferase levels commonly exceed 1000 IU/L, and significant hyperbilirubinemia is often present [22]. Hemorrhagic manifestations range from mucosal bleeding and petechiae to gastrointestinal hemorrhage and hematemesis, resulting from impaired hepatic synthesis, endothelial injury, and coagulation abnormalities [22].

Renal involvement may progress to oliguria and azotemia, frequently occurring alongside hypotension and shock. Acute kidney injury is a common complication in severe cases and may necessitate renal replacement therapy [22]. Neurologic manifestations such as delirium, seizures, and coma may occur due to severe metabolic disturbance and multiorgan failure, while primary encephalitis is uncommon in wild-type infection [23].

Diagnosis

Yellow fever should be suspected in individuals from endemic areas or with recent travel exposure. Diagnosis relies on clinical features, laboratory findings, epidemiologic context, and confirmation by specialized laboratory testing. Definitive diagnosis of yellow fever relies on laboratory or histopathological confirmation. This includes detection of YFV-specific IgM antibodies or a fourfold or greater rise in IgG titers between acute and convalescent sera in the absence of recent vaccination. Viral isolation also establishes the diagnosis. Additional confirmation may be obtained through identification of YFV antigen in tissue by immunohistochemistry or detection of viral genomic sequences in blood or tissue using polymerase chain reaction assays. In fatal cases, characteristic hepatic findings provide supportive evidence. These include midzonal hepatocyte apoptosis with Councilman bodies, steatosis, and minimal inflammatory infiltrate [27].

Clinically, yellow fever frequently remains unrecognized until convalescence or death. A suspected case is defined as an acute febrile illness followed by jaundice within two weeks of symptom onset. Confirmation requires laboratory evidence or an epidemiological link to a laboratory-confirmed case or a recognized outbreak.

Treatment

Yellow fever management is primarily supportive, focusing on organ support and treatment of complications, as no licensed antiviral therapy exists.

Hemodynamic stabilization with fluid resuscitation and vasopressors is essential in shock. Hepatic dysfunction and coagulopathy are treated with blood products when indicated, guided by prothrombin time and INR monitoring. Renal failure may require renal replacement therapy, preferably continuous modalities in unstable patients. Advanced care includes ventilatory support, careful fluid and electrolyte management, nutritional support, and prevention of secondary infections [22].

Experimental and adjunctive treatments have been explored but lack proven efficacy. Liver transplantation has been attempted in select cases of fulminant hepatic failure, but outcomes are limited due to persistent viremia and multiorgan dysfunction [4]. Broad-spectrum antivirals, such as ribavirin, show activity in vitro and in animal models but have not demonstrated consistent clinical benefit in humans [28,29]. Likewise, immunomodulatory approaches targeting cytokine-mediated pathology remain unvalidated and are not recommended for routine use [29].

Early intensive monitoring of patients in the intoxication phase allows timely initiation of transfusions, renal replacement therapy, and vasopressor support, potentially improving outcomes. Current evidence emphasizes that management remains largely supportive, with no disease-specific therapy proven to reduce mortality. The absence of effective antivirals further highlights the importance of preventive measures, especially vaccination and vector control, in reducing disease burden.

Prevention: vaccine and vector control

The YFV was first isolated in 1927, spurring early vaccine development. Initial inactivated vaccines proved inadequate, leading to the adoption of the live attenuated 17D strain [23,24]. Since its introduction in the 1930s, the 17D vaccine has shown high immunogenicity and long-lasting protection, serving as the cornerstone of routine prevention and outbreak control. It induces transient, low-grade viremia in about half of recipients and elicits protective neutralizing antibodies in nearly 99% of vaccinated individuals, with protective immunity typically developing within 10 days (seroconversion rates exceeding 95% among immunocompetent individuals) [6,23]. While boosters were once recommended every 10 years, evidence now indicates that a single dose provides long-lasting, often lifelong immunity, prompting the WHO’s 2013 guidance that a single dose is sufficient for most travelers and residents in endemic regions [24]. Mass vaccination campaigns, alongside routine immunization, remain critical for reducing disease burden and preventing urban outbreaks. High vaccination coverage is crucial for interrupting transmission, reducing spillover from sylvatic cycles, and preventing urban epidemic amplification.

The yellow fever 17D vaccine is a classic example of a live attenuated vaccine, manufactured in embryonated chicken eggs, with each administered dose containing approximately 10⁴-10⁶ plaque-forming units (PFU). Attenuation is achieved through multiple genomic modifications, particularly affecting the envelope (E) protein and nonstructural proteins such as NS2A, thereby reducing viral pathogenicity while maintaining strong immunogenic potential. These alterations consist of defined amino acid substitutions that influence viral replication kinetics, tissue tropism, and immune evasion strategies. Importantly, the vaccine strain demonstrates a high-fidelity replication machinery that restricts viral quasi-species variability and minimizes the likelihood of reversion to virulence, even in the presence of mutagenic pressures such as ribavirin exposure. This polygenic attenuation framework, along with critical residues in envelope domain III that enhance glycosaminoglycan binding and facilitate rapid viral clearance, underlies the vaccine’s long-established safety profile worldwide. From an immunological perspective, vaccination initiates a coordinated immune response characterized by early innate activation, including TNF⁺ neutrophils, IFN-γ-producing natural killer cells, and IL-10⁺ regulatory monocytes, followed by robust adaptive immunity. Strong CD8⁺ T-cell responses are generated, undergoing substantial contraction before maturing into long-lived stem cell-like memory populations. Concurrently, prolonged germinal center reactions lasting six to nine months promote affinity maturation of neutralizing antibodies, resulting in durable humoral immunity that can persist for decades after a single vaccination.

Vector control is a key prevention strategy, targeting both *Ae. aegypti* and sylvatic mosquitoes through environmental management, insecticides, and community efforts to eliminate larval habitats [30]. Integrated vector management, combining chemical, biological, and environmental methods, has been shown to effectively reduce vector populations [30]. Individual protective measures, including the use of insect repellents, protective clothing, and avoidance of peak mosquito activity, are recommended for residents and travelers in endemic or outbreak-prone areas [31].

Surveillance and rapid response underpin comprehensive prevention, including entomologic monitoring, vaccination of at-risk populations, and swift outbreak containment. Global efforts, including the WHO’s Eliminate Yellow Fever Epidemics strategy, focus on protecting vulnerable populations, preventing international spread, and rapidly controlling outbreaks [32]. YFV’s persistence in nonhuman primates and sylvatic mosquitoes makes eradication impossible, making sustained human vaccination crucial to prevent spillover and curb urban transmission [6,22]. Many countries require proof of yellow fever vaccination for travelers, weighing individual risk against the rare chance of serious vaccine-related adverse events [22]. International Health Regulations (IHR) provide the legal framework enabling countries to enforce vaccination requirements for travelers arriving from endemic regions [22].

The details are provided in Table 2, which summarizes yellow fever vaccine types, strains, manufacturers, efficacy, duration of immunity, and contraindications.

**Table 2:** Types of yellow fever vaccination Source: [2,33,34]

Vaccine Type	Strain	Manufacturer	Efficacy	Duration of Immunity	Contraindications
Live Attenuated 17D [33]	17D-204	YF-VAX (Sanofi Pasteur), STAMARIL (Sanofi Pasteur)	95-99% seroconversion after a single dose	Lifelong	Age <6 months (absolute), Age 6-9 months (relative), Pregnancy (relative), Severe immunodeficiency (HIV with CD4 <200, primary immunodeficiency), Thymus disorders, Severe egg allergy
Live Attenuated 17D	17DD	Bio-Manguinhos (Brazil)	95-99% seroconversion	Lifelong	Same as above
Live Attenuated 17D [2]	17D-213	Various African manufacturers	95-99% seroconversion	Lifelong	Same as above
Fractional Dose (Emergency Use) [34]	17D (1/5 standard dose)	Same manufacturers as above, reduced volume	80-90% seroconversion (shorter duration)	Minimum 12 months (not accepted for International Health Regulations (IHR) certificate)	Same as above + not for routine travel certification

Vaccination in travelers

International travel to yellow fever-endemic regions requires careful adherence to vaccination recommendations and to entry regulations governed by the IHR 2005, established by the WHO [2,33]. Yellow fever remains the only disease for which countries are legally permitted to require proof of vaccination as a condition of entry. The disease is endemic in 47 countries across Africa and South and Central America, including 34 African nations and 13 countries in the Americas, where transmission occurs in defined geographic areas. Proof of vaccination is documented through the International Certificate of Vaccination or Prophylaxis (ICVP), the sole internationally recognized certificate, which must be issued by an authorized Yellow Fever Vaccination Center and becomes valid 10 days after primary vaccination [2]. Following the 2016 WHO revision, a single vaccine dose is considered valid for life, provided documentation includes vaccine details, batch number, vaccination date, vaccinator identification, and an official institutional stamp [35].

Yellow fever vaccination requirements for travelers fall into three principal categories. First, vaccination is recommended for personal protection for individuals traveling to endemic regions, particularly rural, forested, or jungle environments where exposure risk is highest [36]. Second, many non-endemic countries require proof of vaccination for travelers arriving from endemic regions to prevent viral importation and local transmission, especially in areas where competent *Ae. aegypti *vectors are present [2]. Third, some countries mandate vaccination for onward travel or transit passengers who have passed through endemic areas, even without leaving airport transit zones, with requirements varying according to transit duration and national policy [2]. Approximately 120 countries maintain entry requirements, particularly across Asia, the Middle East, Oceania, parts of Africa, and Caribbean nations, whereas Europe and North America generally do not impose routine entry requirements (Table 3).

**Table 3:** Vaccination requirements Source: [2,37,38]

Region	Requirement Status	Representative Countries	Key Notes
Asia	Commonly required	India, China, Thailand, Singapore, Malaysia, Indonesia	Often required if arriving from endemic countries; transit exemptions may apply
Middle East	Frequently required	Saudi Arabia, UAE, Qatar, Egypt, Oman	Mandatory for Hajj/Umrah pilgrims from endemic regions
Oceania	Selectively required	Australia, Fiji, Papua New Guinea, Samoa	Usually required if arrival occurs within 6 days of endemic exposure
Africa (non-endemic)	Widely required	South Africa, Morocco, Namibia, Seychelles, Zambia	Enforcement may be strict at land borders and major airports
Caribbean	Required from endemic regions	Barbados, Jamaica, Saint Lucia, Grenada	Preventive measure against importation
Europe	Not routinely required	-	No current routine entry requirements
North America	Not routinely required	USA, Canada, Mexico	Vaccination recommended only for travelers to endemic regions

Implementation and enforcement of vaccination regulations vary considerably among countries. Some nations, including South Africa, Saudi Arabia during the Hajj and Umrah seasons, India, and Tanzania, apply strict enforcement measures, such as entry denial without proof of vaccination (Table 3) [2,37,38]. Other countries demonstrate moderate enforcement with routine certificate checks at major ports of entry, while certain destinations exhibit variable or inconsistent enforcement depending on outbreak activity or local administrative practices. Quarantine measures are generally waived for travelers presenting valid vaccination certificates, although exemptions for infants or individuals with documented medical contraindications may be accepted inconsistently.

Several additional considerations influence travel planning. Fractional vaccine doses administered during outbreak responses do not fulfill IHR certification requirements for international travel. Although WHO guidance recognizes lifelong validity following a single vaccination, some countries may still operate under outdated policies that require booster documentation [35]. Transit regulations also vary widely, with some countries requiring vaccination after brief layovers for travelers originating from endemic areas [38]. Consequently, travelers should verify requirements directly with destination authorities before departure.

Effective pre-travel preparation includes reviewing vaccination requirements for all destinations and transit stops, receiving vaccinations at least 10 days prior to travel, and ensuring vaccinations are administered at an authorized centre. Travelers should carry the original ICVP throughout their journey, maintain backup copies, and obtain official medical exemption documentation where applicable. Vaccination is strongly recommended for travel to endemic areas, even when not mandated for entry, and travel should be postponed during periods of active transmission when vaccination is contraindicated. Finally, travelers should remain aware that policies may change rapidly during outbreaks and should consult official WHO or national travel health guidance prior to departure and before return travel.

Prognosis

Yellow fever outcomes range from full recovery to rapid death in fulminant cases. Prognosis depends mainly on progression to the intoxication phase, development of multiorgan failure, and host factors [22,23]. Severe yellow fever carries a case fatality rate of roughly 20-50%, especially when jaundice, coagulopathy, and hemorrhagic signs reflect advanced systemic involvement [22]. Early prognostic indicators include marked lymphopenia and rapidly rising hepatic transaminases, often preceding clinical deterioration. In hospitalized patients, multiorgan dysfunction, particularly renal failure, shock, severe coagulopathy, and the need for ventilatory or renal support, is strongly associated with mortality [22]. At the population level, outbreaks cause substantial mortality in endemic regions, with the true burden likely underestimated due to underrecognition and incomplete reporting.

## Conclusions

Yellow fever remains a major public health threat in endemic regions of Africa and South America, despite the availability of an effective vaccine. Clinically, it ranges from a self-limited febrile illness to fulminant hepatic failure with hemorrhage and multiorgan dysfunction, driven by intense viral replication, dysregulated immune responses, and complex organ injury mechanisms. Management is primarily supportive, as no antiviral therapy has proven effective. Prevention through vaccination and vector control remains central, with high immunization coverage, integrated vector management, and robust surveillance essential to limiting transmission and outbreaks. Continued research into pathogenesis, prognostic markers, and therapeutic approaches is critical to improving outcomes in severe disease.
